# Novel Bradykinin Receptor Inhibitors Inhibit Proliferation and Promote the Apoptosis of Hepatocellular Carcinoma Cells by Inhibiting the ERK Pathway

**DOI:** 10.3390/molecules26133915

**Published:** 2021-06-26

**Authors:** Yiou Wang, Bingxue Zhang, Yibing Huang, Wenjun Yao, Fei Tao, Yuxin Chen

**Affiliations:** 1 Key Laboratory for Molecular Enzymology and Engineering of the Ministry of Education, School of Life Sciences, Jilin University, Changchun 130012, China; wangyo1994@163.com (Y.W.); zhangbx2016342048@163.com (B.Z.); huangyibing@jlu.edu.cn (Y.H.); 2 Jiangsu ProteLight Pharmaceutical & Biotechnology Co., Ltd., Jiangyin 214437, China; yao.wenjun@protelight.com (W.Y.); tao.fei@protelight.com (F.T.)

**Keywords:** bradykinin receptor inhibitor, hepatocellular carcinoma, apoptosis, bradykinin B1 receptor, ERK signaling pathway

## Abstract

Hepatocellular carcinoma (HCC) is the fifth most common cancer worldwide. Studies have shown that bradykinin (BK) is highly expressed in liver cancer. We designed the novel BK receptor inhibitors J051-71 and J051-105, which reduced the viability of liver cancer cells and inhibited the formation of cancer cell colonies. J051-71 and J051-105 reduced cell proliferation and induced apoptosis in HepG2 and BEL-7402 cells, which may be due to the inhibition of the extracellular regulated protein kinase (ERK) signaling pathway. In addition, these BK receptor inhibitors reversed the cell proliferation induced by BK in HepG2 and BEL-7402 cells by downregulating B1 receptor expression. Inhibiting B1 receptor expression decreased the protein levels of p-ERK and reduced the malignant progression of HCC, providing a potential target for HCC therapy.

## 1. Introduction

Hepatocellular carcinoma (HCC), also known as primary liver cancer, is one of the top five fatal cancers globally and one of the most aggressive and widespread malignancies worldwide [1]. During the early stages of HCC, the symptoms are not obvious and are difficult to detect, however, HCC develops rapidly after the onset of the disease. Once HCC progresses to the advanced stage, the survival rate of patients is generally less than 5% [2]. The onset of liver cancer is a complex and multifactorial process. Excessive alcohol intake, virus infections (hepatitis B or C), and the deterioration of liver cirrhosis can all cause liver cancer [3]. At present, the clinical treatment options are mainly chemotherapy, surgery, and liver transplantation [4]. The molecular targeted drugs regorafenib and sorafenib are used to treat patients with advanced liver cancer [5], however, their efficacy is very low, and their use is associated with significant adverse side effects [6,7]. Similar to other types of cancer, HCC involves the abnormal proliferation of liver cancer cells and cancer cell evasion of apoptosis. HCC also has the ability to form and maintain new blood vessels [8].

Bradykinin (BK), a vasoactive peptide released during inflammation [9], is considered a multipotential stimulant of cancer growth. BK exerts its cellular effects through two pharmacologically distinct G-protein-coupled receptors (GPCRs), the B1 receptor (B1R) and B2 receptor (B2R) [10]. Both BK and its active metabolite Des-Arg^9^-BK can bind to BK receptors. In most cases, B1R expression is absent or very low in normal cells but is upregulated during tissue damage, inflammation, or carcinogenesis [11]. B2R is expressed constitutively with a wide tissue distribution [12]. In special cases, B1R is also present in human neutrophils or some neurons [13]. B1R and B2R are highly expressed in liver cancer cells. Studies show that in trichloroethylene (TCE)-sensitized mice, activation of the kinin-kallikrein system (KKS) [14], especially the activation of B1R, can cause immune-mediated liver injury. However, selectively blocking the B1 receptor prevented this damage [15]. Subsequent studies showed that the use of the B2 receptor antagonist HOE-140 in TCE-damaged liver tissue could inhibit mitogen-activated protein kinase (MAPK) activation as well as reduce the levels of the proinflammatory cytokines interleukin IL-1, IL-6, and tumor necrosis factor (TNF)-α [16]. However, the specific mechanisms of action of BK receptors in liver cancer have not yet been elucidated.

The extracellular regulated protein kinase (ERK) 1/2 signaling pathway is a member of the MAPK signaling pathway family. The ERK 1/2 signaling pathway regulates cell proliferation and plays a pivotal intermediary role in tumor cells. Compared with normal cells, cancer cells and tumor tissues usually evade apoptosis [17]. Moreover, compared with the levels in adjacent normal liver tissues, the ERK signaling pathway is in an abnormally active state of phosphorylation in liver cancer tissues [18]. Highly activated growth factors can activate tyrosine kinase and stimulate the ERK signaling pathway, leading to a cascade reaction and the activation of nuclear transcription factors, such as NF-κB, to promote the proliferation of hepatocellular carcinoma cells [19]. The ERK signaling pathway antagonist PD98059 can promote apoptosis and reduce the levels of p-ERK in liver cancer cells, subsequently decreasing proliferation by inhibiting the activation of the ERK pathway [20].

Currently, BK inhibitors are used to modulate cancer growth. Several peptide hormones (such as BK) have been identified as growth factors for human cancers (lung, prostate, ovarian, gastrointestinal, and breast). Stewart et al. have synthesized a highly potent anticancer peptide mimetic, BKM570, which exhibited impressive growth inhibition of SCLC in vitro and in vivo in nude mice [21]. Based on the structure of BKM570, we designed and synthesized two novel types of BK receptor inhibitors through auxiliary simulation and structural optimization and named them J051-71 and J051-105. In this study, we investigated the inhibitory effects of J051-71 and J051-105 on the proliferation of human HepG2 and BEL-7402 HCC cell lines. We further explored the effect of BK receptor inhibitors on the apoptosis of HCC cells and the underlying mechanisms from the perspective of signaling pathways.

## 2. Results

### 2.1. Design and Synthesis of BK Receptor Inhibitors

**Figure 1:**
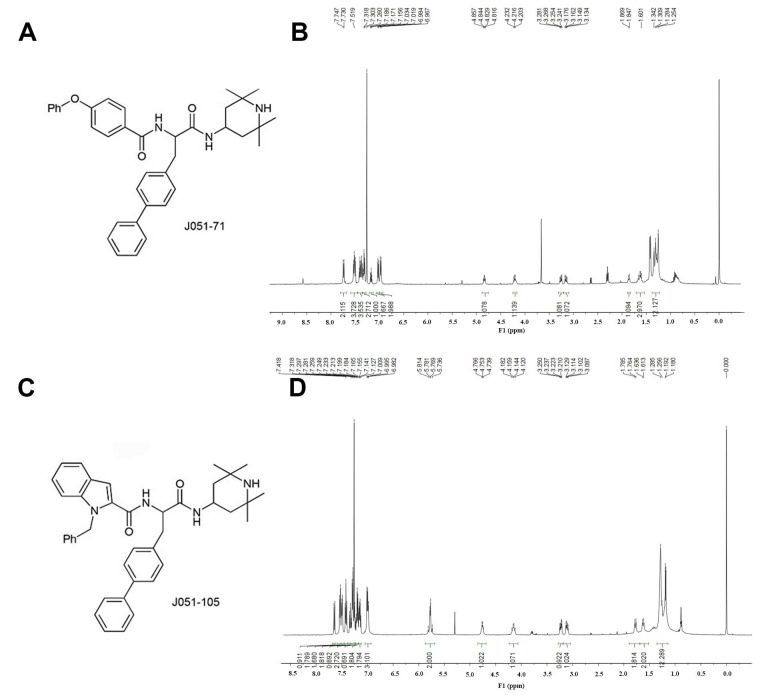
Synthesis of bradykinin receptor inhibitors. (**A**) Structural formula of J051-71. (**B**) NMR hydrogen spectrum of J051-71. (**C**) Structural formula of J051-105. (**D**) NMR hydrogen spectrum of J051-105.

We developed and synthesized the novel and highly effective anticancer peptide simulants J051-71 and J051-105 (Figure 1). Several structural and chemical modifications were performed to generate the *N*-acylated group form of the new BK receptor inhibitors. The hydrophobic groups on the side chain of BKM570 were replaced. Finally, hydrophobic amino groups and amide groups were systematically combined by acylation to generate J051-71 and J051-105. NMR spectroscopy was performed for the structural determination of BK receptor inhibitors. The results of J051-71 were: ^1^H-NMR (500 MHz, CDCl3) δ 7.74 (d, *J* = 8.7 Hz, 2H), 7.53–7.50 (m, 4H), 7.41–7.35 (m, 4H), 7.32 (t, *J* = 7.3 Hz, 3H), 7.17 (t, *J* = 7.4 Hz, 1H), 7.03 (d, *J* = 7.8 Hz, 2H), 6.98 (d, *J* = 8.6 Hz, 2H), 4.86–4.82 (m, 1H), 4.25–4.16 (m, 1H), 3.26 (dd, *J* = 13.5, 6.7 Hz, 1H), 3.16 (dd, *J* = 13.5, 6.7 Hz, 1H), 1.86 (d, *J* = 10.7 Hz, 1H), 1.66–1.60 (m, 3H), and 1.34–1.25 (m, 12H). The nuclear magnetic results of J051-105 were: ^1^H-NMR (500 MHz, CDCl3) δ 7.64 (d, *J* = 8.0 Hz, 1H), 7.53 (d, *J* = 7.5 Hz, 2H), 7.50 (d, *J* = 7.7 Hz, 2H), 7.42 (t, *J* = 7.6 Hz, 2H), 7.34 (d, *J* = 7.3 Hz, 1H), 7.32–7.27 (m, 3H), 7.25–7.23 (m, 1H), 7.21–7.18 (m, 2H), 7.17–7.13 (m, 2H), 7.00–6.98 (m, 3H), 5.78 (q, *J* = 16.5 Hz, 2H), 4.77–4.74 (m, 1H), 4.18–4.12 (m, 1H), 3.25–3.21 (dd, *J* = 13.5, 6.4 Hz, 1H), 3.11 (dd, *J* = 13.5, 7.4 Hz, 1H), 1.77 (d, *J* = 10.4 Hz, 2H), 1.62 (d, *J* = 11.4 Hz, 2H), and 1.29–1.18 (m, 12H). In this study both J051-71 and J051-105 showed the expected quality and had the correct structure.

### 2.2. Anticancer Activity of BK Receptor Inhibitors

After the optimized synthesis, a preliminary activity screening of these compounds was performed. The results confirmed that J051-71 and J051-105 were effective against A549 human lung cancer cells, SGC-7901 human gastric cancer cells, MCF-7 human breast cancer cells, HeLa human cervical cancer cells, MG63 human osteosarcoma cells, and Bx-PC3 human pancreatic cancer cells (Table 1). The new BK receptor inhibitors showed certain inhibitory effects on different cancer cell lines. Moreover, compared with the positive controls: paclitaxel and cisplatin, the anticancer effects of J051-71 and J051-105 were mostly superior to cisplatin and equivalent to paclitaxel. This shows that the novel BK receptor inhibitors have a broad spectrum of anticancer activity.

### 2.3. BK Receptor Inhibitors Inhibit the Cellular Activity of Primary HCC Cells

**Figure 2:**
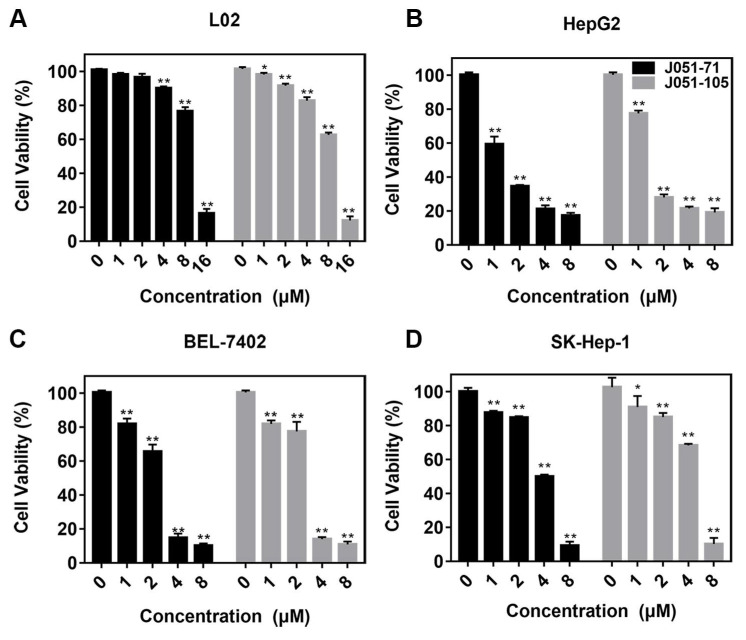
Bradykinin receptor inhibitors inhibit the cell viability of primary hepatocellular carcinoma cells. (**A**) L02, (**B**) HepG2, (**C**) BEL-7402, and (**D**) SK-Hep-1 cells were treated with various concentrations (0, 1, 2, 4, and 8 μM) of J051-71 or J051-105 for 24 h. The cell viability of hepatocellular carcinoma cells was determined by the MTT assay (*n* = 3). * *p* < 0.05, ** *p* < 0.01: the test concentrations (1, 2, 4, and 8 μM) vs. the test concentration (0 μM).

As shown in Figure 2, the decline in the cell viability of human HCC cells was positively correlated with the concentration gradients of J051-71 and J051-105 within a period of 24 h. In addition, J051-71 and J051-105 exhibited different anticancer effects on various cell lines. The cytotoxic effects of J051-71 and J051-105 against HepG2 and BEL-7402 cells were stronger than against SK-Hep-1 cells.

As shown in Table 2, J051-71 and J051-105 inhibited the cell viability of cancer cells at very low concentrations but their effect on normal liver cells was quite weak. Thus, these BK receptor inhibitors are less toxic to normal liver cells at concentrations that are effective against liver cancer cells. Because lower IC_50_ values and more significant effects of J051-71 and J051-105 were observed in HepG2 and BEL-7402 cells, these two HCC cell lines were selected for subsequent experiments.

### 2.4. BK Receptor Inhibitors Inhibit the Proliferation of HCC Cells

**Figure 3:**
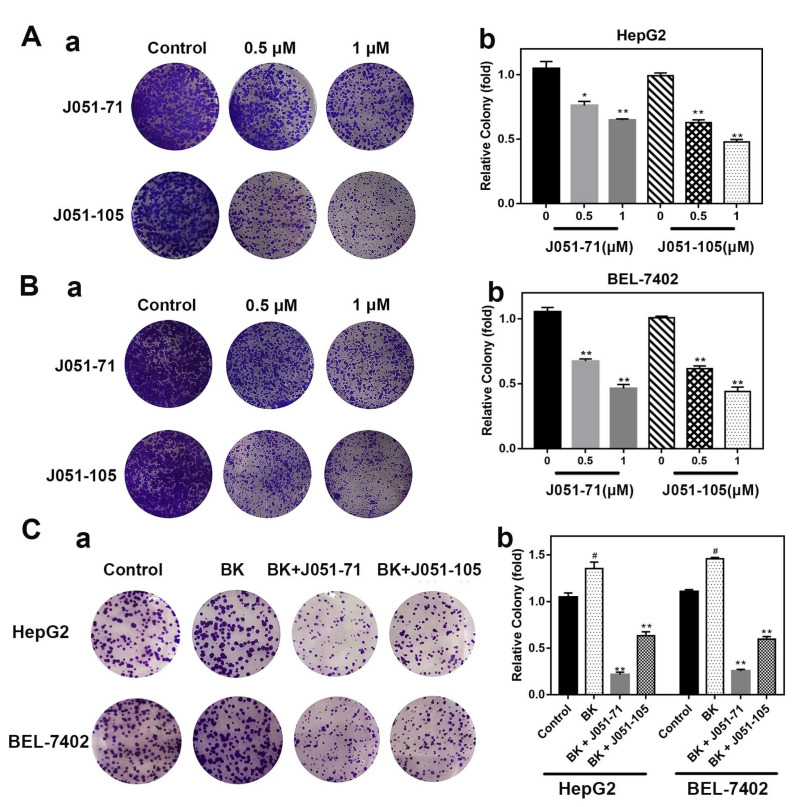
Bradykinin receptor inhibitors inhibit the proliferation of hepatocellular carcinoma cells. (**A**) (**a**) Colonies were stained with crystal violet to evaluate the cell colony formation of HepG2 and BEL-7402 cells. (**b**) Colony formation assay of HepG2 cells treated with various concentrations (0.5 and 1 μM) J051-71 and J051-105 (*n* = 3), HepG2 cells without treatment were used as control. * *p* < 0.05, ** *p* < 0.01 the test concentrations (0.5 and 1 μM) vs. control, respectively. (**B**) (**a**) Colonies were stained with crystal violet to evaluate the cell colony formation of BEL-7402 cells. (**b**) Colony formation assay of BEL-7402 cells treated with various concentrations (0.5 and 1 μM) J051-71 and J051-105 (*n* = 3), BEL-7402 cells without treatment were used as control. * *p* < 0.05, ** *p* < 0.01 the test concentrations (0.5 and 1 μM) vs. control, respectively. (**C**) (**a**) Cells were treated with BK at a concentration of 1 μM and J051-71 and J051-105 at 1 μM. (**b**) Colony formation assay of HepG2 and BEL-7402 cells treated with BK, BK and J051-71 or BK and J051-105 (*n* = 3), HepG2 and BEL-7402 cells without BK treatment were used as control. * *p* < 0.05, ** *p* < 0.01: BK and J051-71 or BK and J051-105 vs. BK. # *p* < 0.05: BK vs. control.

Compared with the control groups, the experimental groups treated with J051-71 and J051-105 exhibited a significantly reduced number of liver cancer cell clones at a drug concentration of 0.5 μM (Figure 3A,B). The quantitative graphs of colony formation experiments (Figure 3A,B) showed that both inhibitors reduced the colony formation rate of HepG2 and BEL-7402 cells.

To further explore the inhibitory effect of J051-71 and J051-105, we added BK as a positive control to induce the proliferation of liver cancer cells and cell colony formation (Figure 3C). J051-71 or J051-105 was added to cells treated with BK (cells without BK treatment were used as a negative control), and the formation of cell colonies was observed. Compared with the negative control, BK clearly stimulated the formation of cell colonies, which was significantly reduced in the presence of J051-71 and J051-105. These results suggest that the BK receptor inhibitors significantly reduced the BK-induced increase in cell colony formation compared with the positive control groups.

### 2.5. BK Receptor Inhibitors Promote the Apoptosis of HCC Cells

**Figure 4:**
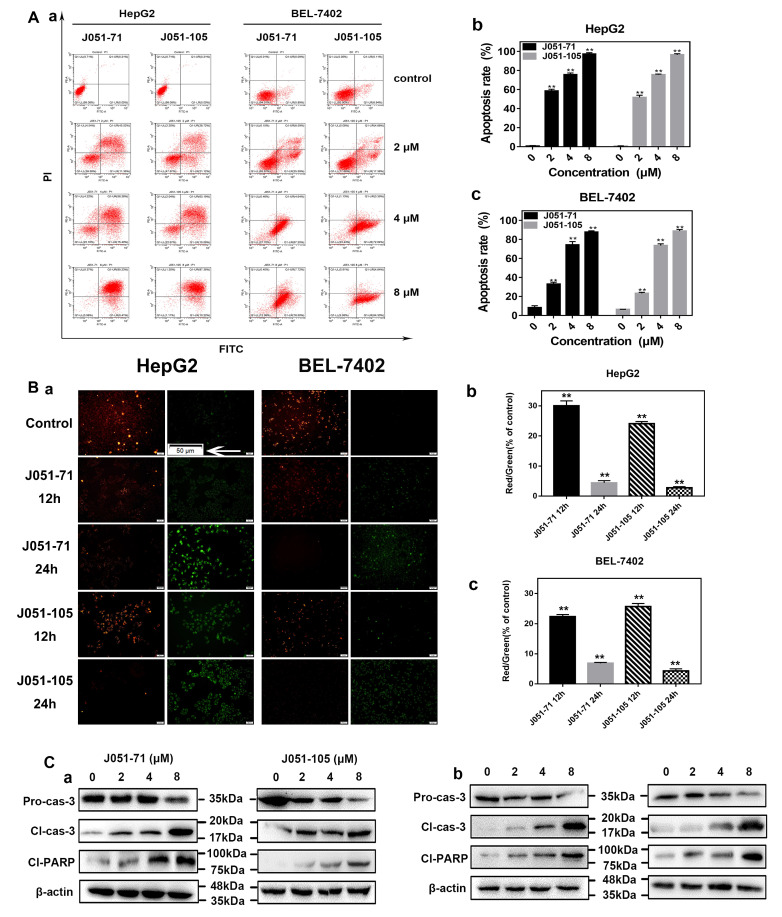
Bradykinin receptor inhibitors promote the apoptosis of hepatocellular carcinoma. (**A**) HepG2 and BEL-7402 cells were exposed to J051-71 or J051-105 (0, 2, 4, and 8 μM) for 24 h (*n* = 3). (**a**). BK inhibitors affect the outcome of hepatocellular carcinoma cell apoptosis. Quantification of apoptotic HepG2 (**b**) and BEL-7402 (**c**) cells, ** *p* < 0.01: the test concentrations (2, 4, and 8 μM) vs. the test concentration (0 μM). (B) HepG2 and BEL-7402 cells were exposed to 2 μM J051-71 alone or 2 μM J051-105 alone for 12 h or 24 h (*n* = 3). (**a**). Qualitative results of fluorescence microscopy of mitochondrial membrane potential. Apoptotic cells were identified by JC-1 staining (green), and non-apoptotic cells were identified by JC-1 staining (red). Scale bars are 50 μm. The calculated percentage ratio of red to green for HepG2 (**b**) and BEL-7402 (c) cells treated with J051-71 and J051-105 for 12h and 24h (*n* = 10), ** *p* < 0.01 vs. control, respectively. (**C**) Western blot analysis of the protein levels of pro-caspase 3, cleaved caspase-3, and cleaved PARP in HepG2 (**a**) and BEL-7402 (**b**) cells (*n* = 3). β-actin served as the loading control.

To investigate the effect of BK receptor inhibitors on the apoptosis of HCC cells, based on the results of cell proliferation, we used J051-71 (1 μM) or J051-105 (1 μM) to treat HepG2 and BEL-7402 cells for 24 h. The effect of apoptosis was not obvious. Then, in order to obtain an obvious apoptotic phenomenon, we treated HepG2 and BEL-7402 cells with different concentrations (such as 2, 4, and 8μM) of J051-71 or J051-105 for 24 h. As shown in Figure 4A(a), both J051-71 and J051-105 increased the apoptosis rate of HepG2 and BEL-7402 cells in a dose-dependent manner. However, the trend of apoptosis induced by the BK receptor inhibitors was different for the two types of HCC cells. With increasing drug concentrations, the total apoptotic rate of HepG2 (Figure 4A(b)) and BEL-7402 (Figure 4A(c)) cells was significantly increased compared with the blank control group. A concentration of 2 μM was selected to detect the changes in mitochondrial membrane potential because it is close to the IC_50_ value. The purpose of measuring the mitochondrial membrane potential is to illustrate the occurrence of premature withering. When premature apoptosis occurs, the mitochondrial membrane potential is high, and vice versa. The JC-1 mitochondrial membrane potential detection kit was used to detect changes in the mitochondrial membrane potential [22]. The lipophilic fluorescent dye JC-1 can freely enter and exit the cell membrane and bind to the mitochondrial matrix, therefore, we could detect the change of mitochondrial membrane potential through the change of fluorescence color. When the mitochondrial membrane potential is high, it will show red fluorescence (excitation wavelength at 490 nm). In contrast, when the mitochondrial membrane potential is low, it will show green fluorescence (excitation wavelength at 525 nm). As shown in Figure 4B(a), whether it was in HepG2 or BEL-7402 cells, compared with the blank control group, the red fluorescence intensity of the inhibitor group decreased with time, and the green fluorescence intensity increased correspondingly. We quantified the amount of red staining and green staining per image using ImageJ analysis software. For BEL-7402 cells, when treated with J051-71 and J051-105 for 12 h, compared to the control, the percentage of red/green ratio was 21.52% and 30.96%, respectively. When treated with J051-71 and J051-105 for 24 h, compared to the control, the percentage of red/green ratio was 5.19% and 4.69% (*p* < 0.01 vs. control), respectively. For HepG2 cells, when treated with J051-71 and J051-105 for 12 h, compared to the control, the percentage of red/green ratio was 29.95% and 23.31%, respectively. When treated with J051-71 and J051-105 for 24 h, compared to the control, the percentage of red/green ratio was 3.60% and 2.49% (*p* < 0.01 vs. control), respectively. From the above results, the decreased percentage ratio of red to green for HepG2 and BEL-7402 cells treated with J051-71 and J051-105 indicated the shift in membrane potential and indirectly indicated cell death (Figure 4B(b,c)). Accordingly, this confirmed that the physiological process of premature cell apoptosis occurred in liver cancer cells when the inhibitor concentration was low [22,23]. Based on the above experimental results, the expression of apoptotic proteins was measured. As shown in Figure 4C, the inhibitors’ dose-dependence increased the protein expression of cleaved caspase-3 and cleaved PARP, whereas the expression of pro-caspase 3 showed the opposite trend. J051-71 and J051-105 exhibited similar effects in the two different HCC cell lines. Together, these results demonstrate that J051-71 and J051-105 up-regulate cleaved caspase-3 expression and PARP cleavage through the caspase pathway and induces the apoptosis of HCC cells.

### 2.6. BK Receptor Inhibitors Reduce the Proliferation of HCC Cells by Inhibiting the ERK Signaling Pathway

**Figure 5:**
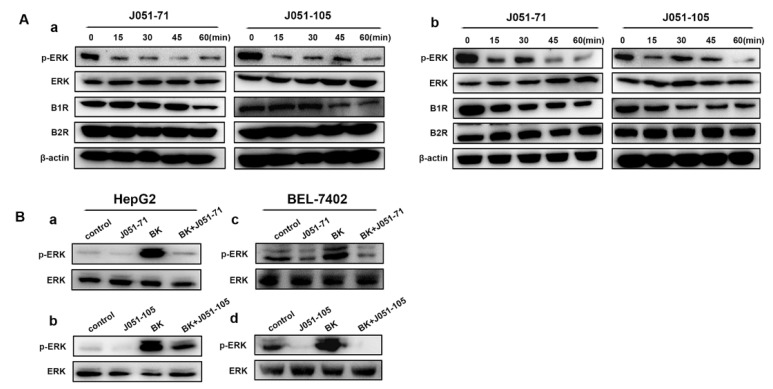
Bradykinin receptor inhibitors reduce the proliferation of hepatocellular carcinoma cells by inhibiting the activation of the ERK signaling pathway. (**A**) HepG2 (**a**) and BEL-7402 (**b**) cells were treated with 2 μM J051-71 and J051-105 for 0, 15, 30, 45, and 60 min (*n* = 3). Protein levels of B1R, B2R, p-ERK, and ERK were determined by Western blot. β-actin served as the loading control. (B) Western blot expression of the indicated proteins in HepG2 cells treated with 2 μM J051-71 (**a**) and J051-105 (**b**) or BEL-7402 cells treated with J051-71 (**c**) and J051-105 (**d**) for 30 min (*n* = 3).

We assessed the phosphorylation of ERK in HepG2 and BEL-7402 cells over time. As shown in Figure 5A(a), HepG2 cells treated with J051-71 and J051-105 exhibited significantly reduced levels of p-ERK within a short time of 15 min. With increasing time, B1R receptor expression in HepG2 cells was also significantly downregulated. In contrast, the expression level of B2R did not change significantly. The trend of Western blot results for BEL-7402 (Figure 5A(b)) cells was similar to HepG2 cells. To further confirm the effect of BK inhibitors on the ERK signaling pathway, we treated liver cancer cells with 10 μM BK for 30 min, added the BK receptor inhibitors to inhibit the effects of BK, and detected the activation of the ERK signaling pathway. In HepG2 or BEL-7402 cells, the presence of BK significantly induced the expression of p-ERK, whereas J051-71 and J051-105 significantly inhibited this increased phosphorylation (Figure 5B).

## 3. Discussion

In recent years, the types of BK receptor inhibitors have evolved from peptides with natural amino acids to peptidomimetics and non-peptides. A variety of BK receptor inhibitors have been synthesized and achieved experimental progress [24]. Studies have shown that in lung and prostate cancer, BKM570 has enhanced growth inhibitory effects compared with conventional chemotherapeutic drugs and significant tumor-suppressive effects in vivo [25]. As a promising multi-targeted anticancer compound, it can selectively stimulate the apoptosis of cancer cells [26]. In the study of glioblastoma, BKM570 also strongly inhibited the phosphorylation of ERK 1/2 and activation of the Akt signaling pathway to reduce the cell viability of glioblastoma cells. As a result, BKM570 is expected to become a new potential anticancer drug [27]. Other studies have shown that BKM570 can have cytotoxic effects similar to cisplatin on cell lines derived from epithelial ovarian cancer (EOC), and it can also synergize with cisplatin to inhibit cell growth. In addition, BKM570 has an effective antiproliferative mechanism [28]. BKM570 can downregulate cellular activities, including cell growth metabolism, cell cycle regulation, inflammation and immune responses, signal transduction, protein biosynthesis, transcription regulation, and transport.

Non-peptide BK inhibitors show significant anticancer activities in both in vitro and in vivo cancer models. In this study, we designed the novel BK receptor inhibitors J051-71 and J051-105. They are more stable in structure and have a wider range of anticancer activities. J051-71 and J051-105 inhibited the growth of HepG2 and BEL-7402 HCC cells in a concentration-dependent manner. Further, J051-71 and J051-105 inhibited the activation of the ERK signaling pathway by reducing the expression of B1R. As B1R is widely expressed in cancer cells and inflammatory tissues, the inhibition of B1R expression would likely target and reduce the proliferation of cancer cells.

Based on previous studies, inhibiting related pathways, or suppressing BK receptors could impair the BK-mediated promotion of cell growth [29]. As shown in Figure 5A, when cells were treated with an inhibitor in the absence of BK, p-ERK decreased over time. This phenomenon may be due to one of the three following reasons: (1) BK receptors may perform a secondary function independent of BK that supports cancer cell proliferation and survival; (2) cell lines used in this study may produce BK and respond in a cell-autonomous manner in cell culture; (3) inhibitors are non-specifically toxic. BK is generally secreted by the liver and kidneys in the body, and there is little research on whether normal cultured cancer cells secrete bradykinin. It is difficult to determine the concentration of bradykinin in the culture medium. Regarding the third possibility, although there is a high inhibitory effect on the proliferation of normal cells at high concentrations, the concentration of inhibitors we chose to act on cancer cells is relatively small. Under the action of low concentration the inhibitory effect on L02 is not significant. Hence, low concentrations were used to explore the effect of inhibitors on liver cancer cells. In view of these reasons, it seems that the BK receptor may have a secondary function, which is independent of BK-induced signal transport. Follow-up verification is required to conduct further in-depth research.

In this study, in order to evaluate the effect of J051-71 and J051-105, the cell lines with high expression of B1R and B2R were utilized. Several cell lines, such as human lung cancer cell A549, normal liver cell line L02, three hepatocarcinoma cell lines SK-Hep-1, HepG2 and BEL-7402, human pancreatic cancer Bx-PC3, and human breast cancer cell MCF-7 were used to measure the expression of B1R and B2R. As shown in Figure S1 (Supplementary Materials), it is clear to see that the hepatocarcinoma cells HepG2 and BEL-7402 show a higher protein expression than the normal liver cells L02. Therefore, the hepatocarcinoma cells HepG2 and BEL-7402 were selected to perform the follow-up research. Different doses of the novel BK receptor inhibitors J051-71 and J051-105 exhibited broad-spectrum anticancer activity and significantly inhibited the growth of carcinoma cells. Both BK receptor inhibitors reduced B1R protein expression in liver cancer cells but had no significant effect on B2R expression. In addition, J051-71 and J051-105 inhibited the abnormal phosphorylation in response to BK-induced over-activation of the ERK1/2 signaling pathway. Therefore, we speculated that the novel BK receptor inhibitors inhibited the activation of the ERK1/2 signaling pathway mediated by B1R, thereby affecting the proliferation and growth of the HCC cells. We found that these promising multi-target anticancer compounds selectively stimulated apoptosis and also inhibited cell proliferation of the HCC cells. In addition, these BK inhibitors have the advantage of being combined with other chemotherapeutic agents to enhance their anticancer activity.

## 4. Materials and Methods

### 4.1. Materials

(Benzotriazol1yloxy) tris (dimethylamino) phosphonium hexafluorophosphate (BOP), Boc-4-phenyl-Phe-OH (BOC), 2-(7-azabenzotriazol-1-yl)-*N*,*N*,*N*′,*N*′-tetramethyluronium hexafluorophosphate (HATU), *N*,*N*-diisopropylethylamine (DIPEA), 4-amino-2,2,6,6-tetramethylpiperidine, 4-phenoxybenzolc acid, and methyl indole-2-carboxylate were purchased from Aladdin (Shanghai, China). CH_3_CN, trifluoroacetic acid (TFA), tetrahydrofuran (THF), *N*,*N*-dimethylformamide (DMF), methanol (MeOH), dichloromethane (DCM), HBr, HCl, ethyl acetate (EtOAc), acetic acid (HOAc), and petroleum ether (PE) were purchased from Beijing Chemical Works (Beijing, China). MTT was purchased from Sigma-Aldrich (St. Louis, MO, USA). The FITC Annexin V/PI apoptosis detection kit was purchased from BestBio (Shanghai, China), and the JC-1 mitochondrial membrane potential detection kit was purchased from BestBio (Shanghai, China). Anti-pro caspase-3, anti-cleaved caspase-3, BDKRB1, BDKRB2, anti-ERK1/2, and anti-phospho-ERK1/2 antibodies were purchased from Cell Signaling Technology (Boston, MA, USA). The human liver cancer cell lines HepG2, BEL-7402, and SK-Hep-1, and human normal liver cell line L02 were obtained from the Institute of Biochemistry and Cell Biology (IBCB), Chinese Academy of Sciences (Shanghai, China).

### 4.2. Synthesis of BK Receptor Inhibitors

The BK receptor inhibitors J051-71 and J051-105 were separated and purified by silica gel column chromatography. BOP, BOC, 4-amino-2,2,6,6-tetramethylpiperidine, and DIPEA were added to dry CH_3_CN (50 mL) and reacted overnight. The organic layer was separated by a 1:1 H_2_O/EtOAc mixed solution. After dry extraction with anhydrous MgSO_4_, the extraction was eluted with 10:1 DCM/MeOH and separated by silica gel column chromatography. The Rf value was 0.82. For BOC cracking, the reactant was added to a 1:4 TFA/DCM reaction system and stirred at room temperature for 30 min. The BOC protective base was removed by TFA. A saturated HCl/EtOAc solution was added dropwise under ice bath conditions and stirred for 10 min. Following concentration under reduced pressure, the product was obtained as a white solid.

The reaction products, carboxylic acid, HATU, and DIPEA were added to dry DMF and stirred at room temperature for 24 h. DCM was used to extract the reactants, and the washing and the drying steps were repeated. The carboxylic acid used in the synthesis of J051-71 was 4-phenoxybenzoic acid. DCM/MeOH (10:1) was used for elution, and the Rf value of the pure product was 0.63. The carboxylic acid required for J051-105 was made in our laboratory. Methyl indole-2-carboxylate, HBr, and NaH were dissolved in THF and stirred at room temperature for 48 h under nitrogen protection. The products after decompression and concentration were prepared by aldehyde-alcohol condensation in the presence of EtOH at room temperature for 12 h after the addition of LiOH. Finally, the carboxylic acid needed for the synthesis of J051-105 was obtained after extraction and drying.

Other preparation methods were consistent with the steps of J051-71 synthesis. The pure products were separated with PE: EtOAc (10:1), and the Rf value of the pure products was 0.55. The compounds were characterized by nuclear magnetic resonance (NMR).

### 4.3. Cell Culture, Cell Viability, and Proliferation Assays

Three liver cancer cell lines (HepG2, BEL-7402, and SK-Hep-1) and normal liver cells (L02) were cultured in Dulbecco’s Modified Eagle Medium (DMEM) supplemented with 10% heat-inactivated fetal bovine serum (FBS) (Kangyuan Biotechnology Co., Tianjin, China), 100 mg/mL penicillin, and 250 mg/mL streptomycin. The cultures were maintained at 37 °C in a 5% CO_2_ incubator.

Cell viability was measured using the tetrazolium colorimetric (MTT) method. Cells were seeded in a 96-well culture plate at a density of 1 × 10^4^ cells/well. After incubation for 12 h, the cells were treated with different concentrations of J051-71 (0, 1, 2, 4, and 8 μM) or J051-105 (0, 1, 2, 4, and 8 μM) for 24 h. Then, 20 μL MTT was added to each well and incubated at 37 °C with 5% CO_2_ in an incubator for 4 h. The medium was then replaced with 150 μL of dimethyl sulfoxide (Sigma-Aldrich, St. Louis, MO, USA) for 10 min to dissolve the formazan crystals. The OD values were measured at 492 nm by a microplate reader (Infinite F200 Pro, TECAN, Männedorf, Switzerland) and normalized to the control. The cell viability and 50% inhibitory concentration (IC_50_) were calculated with Origin 8.0 software (OriginLab, Northampton, MA, USA).

### 4.4. Colony Formation

The cell population formed by the progeny of a single cell that proliferated for more than six generations in vitro became a colony. These cell clones can demonstrate the strength of cancer cell proliferation. To determine the viability of liver cancer cells, the colony formation rate was measured. Cells (500 cells/well in 100 μL medium) were plated into 6-well plates and treated with J051-71 or J051-105 at various concentrations (0, 0.5, and 1 μM) for two weeks. The concentration used in the experimental group with BK added was 1 μM. The order of action of the experimental group drugs co-incubated with BK and the new bradykinin receptor inhibitor was: 1 μM BK acted for half an hour, and then 1μM J051-71 or J051-105 was added to treat the cells. HepG2 and BEL-7402 cells not treated with bradykinin inhibitor or BK were used as controls. After incubating the cells in 4% PFA for 10 min, they were stained with 0.25% crystal violet (Solarbio, Beijing, China) for 15 min. More than 50 cell clones were counted under the microscope, and then the colony formation rate was calculated according to the following formula:

colony formation rate (%) = (colony number/number of inoculated cells) × 100(1)

### 4.5. Annexin V/PI Double Staining

For the apoptosis assay, Annexin V-FITC and propidium iodide (PI) apoptosis detection kits were used, and apoptosis was detected by flow cytometry (FACSCalibur, Becton-Dickinson, San Jose, CA, USA). Cells were seeded in 6-well plates at a density of 3 × 10^5^ cells/well. After 12 h incubation, the cells were treated with different concentrations (0, 2, 4, and 8 μM) of J051-71 or J051-105 for 24 h. Then, the cells were collected, washed with phosphate-buffered saline solution, and stained with 5 μL of Annexin V-FITC and 5 μL of PI for 15 min. Finally, the fluorescence was measured using flow cytometry.

### 4.6. Measurement of Mitochondrial Membrane Potentials

The JC-1 mitochondrial membrane potential detection kit was used to detect changes in the mitochondrial membrane potential. HepG2 and BEL-7402 cells were seeded in 6-well plates. After 12 h incubation, the cells were treated with J051-71 (2 μM) or J051-105 (2 μM) for 12 h and 24 h, respectively. Then, cells were incubated with a JC-1 staining solution for 15 min in the dark, and images were obtained with a fluorescence microscope (Olympus, Tokyo, Japan). The ImageJ software was used to quantify the red and green fluorescence intensity for the obtained fluorescence images. Then the fluorescence intensity ratio of red and green was calculated for control, J051-71 and J051-105, respectively. Finally, the relative fluorescence intensity ratio of red and green of the J051-71 and J051-105 groups was calculated compared with the control [23].

### 4.7. Western Blot Analysis

The cells were collected and total proteins were extracted with phenylmethanesulfonyl fluoride and RIPA buffer (50 mM Tris-HCl pH 7.4, 150 mM NaCl, 1% Triton X-100, 1% sodium deoxycholate, and 0.1% SDS) in a ratio of 1:100. The protein concentration in each sample was determined by the BCA method and adjusted to be consistent. To completely denature the protein, 5× SDS-PAGE protein loading buffer (1 M Tris-HCl pH 6.8, 10%SDS, 25 mg BPB, and 2.5mL glycerin) was added to protein samples, which were boiled at 100 °C for 5 min. The protein in the cell lysate was separated with 10% SDS-PAGE and then transferred to a PVDF membrane. The PVDF membrane was blocked with 5% skimmed milk powder for 1 h and incubated with the indicated primary antibody (1:1000) overnight at 4 °C. After washing with TBST (10×, 200 mL (100 mM Tris-Hcl + 1500 mM NaCl), 5 mL (20% Tween-20)), the membrane was incubated with the horseradish peroxidase-conjugated goat anti-rabbit IgG secondary antibody for 1 h. Western blotting was performed using a chemiluminescence substrate (Thermo Scientific, MA, USA), and protein signals were detected using the Tanon 2500 chemiluminescence imaging system (Shanghai, China), as shown in Figure 4 and Figure 5 and Figure S1.

### 4.8. Statistical Analysis

The data are expressed as the average of at least three independent experiments, and statistical significance was analyzed by one-way ANOVA using GraphPad Prism 5.0 software (San Diego, CA, USA). *p* < 0.05 was considered statistically significant.

## 5. Conclusions

In this study, the novel peptidomimetic BK receptor inhibitors were obtained by covalently coupling the hydrophobic amino and amide groups. At the effective doses, the new BK receptor inhibitors showed a weak effect on normal cells but exhibited significant inhibitory effects against cancer cells. J051-71 and J051-105 significantly reduced the proliferation ability of hepatoma cells, which may be due to the inhibition of the activation of the ERK signaling pathway. Additionally, both J051-71 and J051-105 induced the apoptosis of HCC cells through the intrinsic caspase apoptotic pathway. The efficacy of BK receptor inhibitors in HCC provides novel insights for the development of new therapeutics against liver cancer with potent anticancer activities.

## Figures and Tables

**Table 1:** IC_50_ values of J051-71, J051-105, paclitaxel, and cisplatin against different cancer cells (*n* = 6).

Cell Line	J051-71 (μM)	J051-105 (μM)	Paclitaxel (μM)	Cisplatin (μM)
A549	1.28	2.33	0.90	9.38
SGC-7901	5.07	5.49	9.05	12.4
MCF-7	2.20	2.47	<2.0	8.20
HeLa	5.75	5.00	12.30	3.41
MG63	3.22	3.46	<2.0	23.17
Bx-PC3	2.11	2.99	15.27	20.83

**Table 2:** IC_50_ of J051-71 and J051-105 in normal liver cells L02 and liver cancer cells HepG2, BEL-7402 and SK-Hep-1 (*n* = 6).

Cell Line	J051-71 (μM)	J051-105 (μM)
L02	9.53	10.33
HepG2	1.38	1.53
BEL-7402	2.53	2.76
SK-Hep-1	4.78	5.86

## Data Availability

The datasets supporting the conclusions of this article are included within the article.

## Supplementary Materials

The following are available online, Figure S1: The protein expression of B1R and B2R in human lung cancer cell A549, normal liver cell line L02, three hepatocarcinoma cell lines SK-Hep-1, HepG2 and BEL-7402, human pancreatic cancer Bx-PC3 and human breast cancer cell MCF-7 by Western blot.

## Sample Availability

Samples of the compounds are not available from the authors.

## Abbreviations

HCC	Hepatocellular carcinoma
BK	bradykinin
GPCRs	G-protein-coupled receptors
B1R	B1 receptor
B2R	B2 receptor
KKS	kinin-kallikrein system
TCE	trichloroethylene
MAPK	mitogen-activated protein kinase
IL	interleukin
TNF	tumor necrosis factor
BOC	Boc-4-phenyl-Phe-OH
HATU	hexafluorophosphate
DIPEA	diisopropylethylamine
TFA	trifluoroacetic acid
THF	tetrahydrofuran
MeOH	methanol
DCM	dichloromethane
EtOAc	ethyl acetate
PE	petroleum ether
IBCB	Institute of Biochemistry and Cell Biology
NMR	nuclear magnetic resonance
DMEM	Dulbecco’s Modified Eagle Medium
FBS	fetal bovine serum
PI	propidium iodide
