# Supplementary material for: Novel Bradykinin Receptor Inhibitors Inhibit Proliferation and Promote the Apoptosis of Hepatocellular Carcinoma Cells by Inhibiting the ERK Pathway
Source: Molecules. 2021 Jun 26;26(13):3915. doi: 10.3390/molecules26133915 (PMC8272207; doi:10.3390/molecules26133915)
Supplement: Supplementary file 1 [file molecules-26-03915-s001.zip › molecules-1234535-supplementary.pdf]

Novel Bradykinin Receptor Inhibitors Inhibit Proliferation and Promote the Apoptosis of Hepatocellular Carcinoma Cells by Inhibiting the ERK Pathway

Yiou Wang [1], Bingxue Zhang [1], Yibing Huang [1], Wenjun Yao [2], Fei Tao [2] and Yuxin Chen [1,2,*]

[1] Key Laboratory for Molecular Enzymology and Engineering of the Ministry of Education, School of Life Sciences, Jilin University, Changchun 130012, China; wangyo1994@163.com (Y.W.); zhangbx2016342048@163.com (B.Z.); huangyibing@jlu.edu.cn (Y.H.)

[2] Jiangsu ProteLight Pharmaceutical & Biotechnology Co., Ltd., Jiangyin 214437, China; yao.wenjun@protelight.com (W.Y.); tao.fei@protelight.com (F.T.)

* Correspondence: chen_yuxin@jlu.edu.cn; Tel.: +86-431-85155200

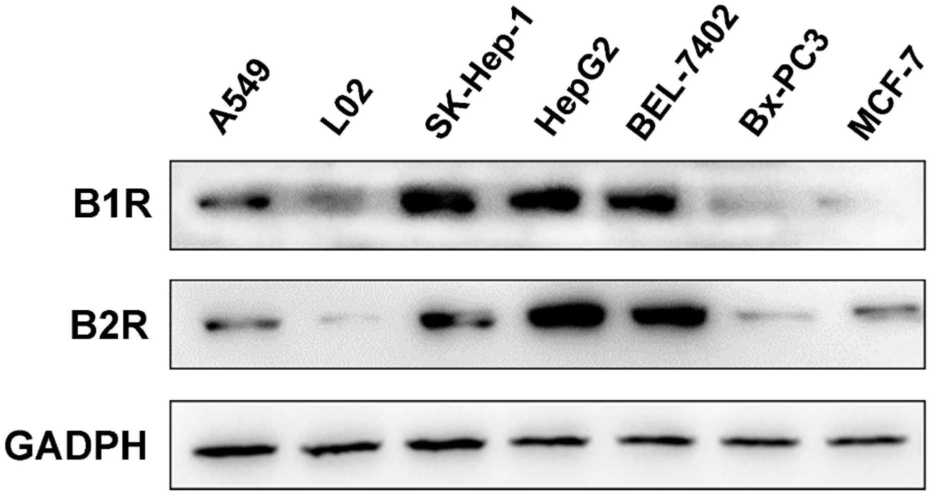

Figure S1. The protein expression of B1R and B2R in human lung cancer cell A549, normal liver cell line L02, three hepatocarcinoma cell lines SK-Hep-1, HepG2 and BEL-7402, human pancreatic cancer Bx-PC3 and human breast cancer cell MCF-7 by Western blot. GADPH was used as a loading control. .
